# Supplementary material for: Neural activity profiles reveal overlapping, intermingled subpopulations spanning area borders in mouse sensorimotor cortex
Source: eLife. 2026 Jul 3;14:RP109240. doi: 10.7554/eLife.109240 (PMC13331482; doi:10.7554/eLife.109240)
Supplement: Peer review history

# Editor report: eLife Assessment

**DOI:** 10.7554/eLife.109240.3.sa0

**Authors:** Brice Bathellier

**Affiliations:** Centre National de la Recherche Scientifique France

**Keywords:** Compelling, Fundamental

This **fundamental** study provides **compelling** evidence for the functional segregation of the sensorimotor cortex into precisely delineated areas, and highlights a rapid transition in functional properties at the boundaries between these areas. This result further confirms and extends recent work on the diversity of neural response specificities across cortical areas in the context of complex behavioral tasks. This work will be of interest to neuroscientists studying sensory-motor functions.

# Referee report: Reviewer #1 (Public review)

**DOI:** 10.7554/eLife.109240.3.sa1

Summary:

Here the authors address the organization of reach-related activity in layer 2/3 across a broad swath of anterodorsal neocortex that included large subregions of M1, M2, and S1. In mice performing a novel variant water-reaching task, the authors measured activity using two-photon fluorescence imaging of a GECI expressed in excitatory projection neurons. The authors found a substantial diversity of response patterns using a number of metrics they developed for characterizing the PETHs of neurons across reach conditions (target locations). By mapping single-neuron properties across cortex, the authors found substantial spatial variation, only some of which aligned with traditional boundaries between cortical regions. Using Gaussian mixture models, the authors found evidence of distinct response types in each region, with several types prominent in multiple cortical regions. Aggregating across regions, four primary subpopulations were apparent, each distinct in their average response properties. Strikingly, each subpopulation was observed in multiple regions, but subpopulation members from different regions exhibited largely similar response properties.

Strengths:

The work addresses a fundamental question in the field that has not previously been addressed at cellular resolution across such a broad cortical extent. I see this as truly foundational work that will support future investigation of how the rodent brain drives and controls reaching.

The quantification is thoughtful and rigorous. It is great that the authors provide explanation for and intuition behind their response metrics, rather than burying everything in the Methods.

The Discussion and general contextualization of the Results is thorough, thoughtful, and strong. It is great that the authors avoid the common over-interpretation of classical observations regarding cortical organization that are endemic in the field.

All things considered, this is the best paper regarding spatial structure in the motor system I have ever read. The breadth of cellular resolution activity measurement, the rigor of the quantification, and the clear and open-minded interrogation of the data collectively have produced a very special piece of work.

Weaknesses:

There are two important issues left unaddressed that the authors plan to address in their future work. The first is the relation between observed neural activity patterns and movement kinematics, and in particular how much the activity variation across target locations may relate to the kinematic differences across these different conditions, as opposed to true higher-order movement features like reach direction. The second issue is how to interpret the results in relation to existing ideas about behavioral organization in motor/premotor cortex.

Comments on revised version:

The authors have done an excellent job addressing my previous concerns. I have no additional concerns with the manuscript.

# Referee report: Reviewer #2 (Public review)

**DOI:** 10.7554/eLife.109240.3.sa2

Summary:

The functional parcellation of cortical areas is a critical question in neuroscience. This is particularly true in frontal areas in mice. While sensory areas are relatively well characterized by their tuning to sensory stimuli, the situation is much less clear for motor areas. This has become even more ambiguous since recent studies using large-scale neuronal recordings consistently report mixed sensory and motor-related activity throughout the brain and motor mapping studies have shown that movements evoked by cortical stimulation are by no means limited to motor areas alone. Here, the authors use a correlation approach combining large-scale functional imaging at cellular-resolution with movement-tracking in mice executing a reaching task. Across multiple recording sessions in the same animals, the authors have imaged a large portion of the sensorimotor cortex at cellular resolution in mice performing a reaching task, recording the activity of nearly 40,000 neurons. By aligning the calcium signal of each neuron to three task events-the Go cue triggering the reach, the onset of paw lift, and the contact between the paw and the target-for different target positions, the authors identified different response patterns distributed differently across cortical areas. They defined a set of features that describe the neurons' response pattern, representing the temporal dynamics and tuning properties for the different target positions. These features were used to construct cortical maps, and the authors show that, interestingly, gradient maps obtained from the first derivative of the feature maps reveal sharp discontinuities at the boundaries between anatomically defined cortical areas. Using dimensionality reduction of the neuronal response features, the authors found that, despite clear differences in their average response properties, individual neurons from the same cortical areas do not form distinct clusters in the reduced-dimensional space. In fact, most areas contain heterogeneous neuronal populations, and most neuronal populations are present in multiple areas, albeit in different proportions. Interestingly, the authors identified four neuronal subpopulations based on the distance between the components of the Gaussian mixture model used to model the distribution of neurons within each area. One of these subpopulations is almost exclusively represented in the anterior M2 cortex, while another is broadly distributed across the different areas.

Strengths:

This article is based on an impressive dataset of nearly 40,000 neurons covering a large portion of the sensorimotor cortex and on innovative analytical approaches. This study is likely the first to clearly demonstrate boundaries between cortical areas defined based on the responses of individual neurons. This innovative approach to functional mapping of cortical areas potentially opens up new perspectives for higher-resolution mapping of frontal cortical areas, using a broader repertoire of sensory and motor evoked responses.

Weaknesses:

One limitation of this study - inherent in most cell imaging studies - is that it only takes into account the activity of neurons in superficial cortical layers. One might think that taking into account neuronal activity across the different layers would allow for an even finer functional cortical segmentation.

Comments on revised version:

The authors have answered all my questions and this new version has largely improved in clarity.

# Author comment: Author response

**DOI:** 10.7554/eLife.109240.3.sa3

**Authors:** Sohrab Salimian, Harrison Grier, Matthew Tyler Kaufman

**Affiliations:** University of Chicago Chicago United States; University of Chicago Chicago United States; University of Chicago Chicago United States

The following is the authors’ response to the original reviews.

In preparation for release of the analysis code used in the paper, we made many analyses more parallel to one another in their exact preprocessing. This resulted in very slight changes to many panels, but these changes are nearly invisible and conclusions did not change. In one case, though, we realized that the way we were presenting data was potentially misleading (the timing plot in Figure 3A). The original plot was of the distribution of pixel values from the spatially smoothed map instead of distributions over individual neurons. We have now swapped it out for better interpretability and changed the accompanying text accordingly.

**Public Reviews:**

**Reviewer #1 (Public review):**

Summary:

Here, the authors address the organization of reach-related activity in layer 2/3 across a broad swath of anterodorsal neocortex that included large subregions of M1, M2, and S1. In mice performing a novel variant water-reaching task, the authors measured activity using two-photon fluorescence imaging of a GECI expressed in excitatory projection neurons. The authors found a substantial diversity of response patterns using a number of metrics they developed for characterizing the PETHs of neurons across reach conditions (target locations). By mapping single-neuron properties across the cortex, the authors found substantial spatial variation, only some of which aligned with traditional boundaries between cortical regions. Using Gaussian mixture models, the authors found evidence of distinct response types in each region, with several types prominent in multiple cortical regions. Aggregating across regions, four primary subpopulations were apparent, each distinct in its average response properties. Strikingly, each subpopulation was observed in multiple regions, but subpopulation members from different regions exhibited largely similar response properties.

Strengths:

The work addresses a fundamental question in the field that has not previously been addressed at cellular resolution across such a broad cortical extent. I see this as truly foundational work that will support future investigation of how the rodent brain drives and controls reaching.

The quantification is thoughtful and rigorous. It is great that the authors provide an explanation for and intuition behind their response metrics, rather than burying everything in the Methods.

The Discussion and general contextualization of the results are thorough, thoughtful, and strong. It is great that the authors avoid the common over-interpretation of classical observations regarding cortical organization that are endemic in the field.

All things considered, this is the best paper regarding spatial structure in the motor system I have ever read. The breadth of cellular resolution activity measurement, the rigor of the quantification, and the clear and open-minded interrogation of the data collectively have produced a very special piece of work.

Thank you! We really, really appreciate this!

Weaknesses:

The behavioral task is very impressive and an important contribution to the field in its own right. However, given that it appears substantially different from the one used in the previous paper, the characterization of the behavior provided in the Results is too brief. More illustration of the behavior would be helpful. For example, it is rather deep into the paper when the authors reveal that the mice can whisk to help localize the target location. That should be expressed at the outset when the behavior is first described. Other suggestions for elaborating the behavior description are included below.

Thank you. Although the task will be treated in greater detail in the next paper (where we more closely relate neural activity to the kinematics), we have added more exposition of the task here. In particular, we now include a figure with a characterization of the trial-to-trial variability across reaches to the same target versus across reaches to different targets (Figure 2-figure supplement 1B). This supports the idea that the mice aimed their reaches. We have also expanded that text.

Regarding whisking, we have now revised that text to make clear that we do not know how the mice localize the spout. The original work by Galinanes and Huber argued that they find the spout by sniffing the water; they may do the same here, or may find it via whisking. It is also possible that the whisking they do is simply because the spout moves in and they are excited, or startled, or do it by reflex. We simply have no evidence one way or another. We have therefore revised the text to make it clearer that whisking-related activation could have occurred for a variety of reasons.

Statistical support for key claims is lacking. For example, "The five areas of interest varied in the fraction of neurons that were modulated: M2 had 14%, M1 had 23%, S1-fl had 30%, S1-hl had 25%, and S1-tr had 27%" - I cannot locate the statistical tests showing that these values are actually different. Another example is Figure 7, where a key observation is that distributions of PETH features are distinct across regions. It is clear that at least some distributions are not overlapping, but a clearer statistical basis for this key claim should be provided.

Good idea. For the proportions, we have now added first a Chi-square test for homogeneity to show that there is variation in the proportions, then shown the results of pairwise two-proportion Z tests (Bonferroni-corrected for multiple comparisons) as a binary matrix in Figure 3-figure supplement 1B. For the area distributions in the t-SNE space (Figure 7), we have added a 2-dimensional Kolmogorov-Smirnov test, again corrected for multiple comparisons, with p-values quoted in the text.

I understand that the authors are planning a follow-up study that addresses the relation between activity patterns and kinematics. One question about interpreting the results here though, is how much the activity variation across target locations may relate to the kinematic differences across these different conditions, as opposed to true higher-order movement features like reach direction.

We agree this is a very important question. However, having done many of the analyses to examine the question for the next paper in the series, we do not know of a shortcut to the right answer. This question requires thorough treatment, and so we leave it to be covered in subsequent work. Instead, after our speculation about how responses suggest function, we are now explicit that these hypotheses needs testing:

“In each of these cases, determining the relationships of the observed activity patterns to function will require specific attempts to link the activity to kinematics, target location, sensory feedback, and more; these relationships will be addressed in future work.”

**Reviewer #2 (Public review):**

Summary:

The functional parcellation of cortical areas is a critical question in neuroscience. This is particularly true in frontal areas in mice. While sensory areas are relatively well characterized by their tuning to sensory stimuli, the situation is much less clear for motor areas. This has become even more ambiguous since recent studies using large-scale neuronal recordings consistently report mixed sensory and motor-related activity throughout the brain, and motor mapping studies have shown that movements evoked by cortical stimulation are by no means limited to motor areas alone. Here, the authors use a correlation approach combining large-scale functional imaging at cellular resolution with movement-tracking in mice executing a reaching task. Across multiple recording sessions in the same animals, the authors have imaged a large portion of the sensorimotor cortex at cellular resolution in mice performing a reaching task, recording the activity of nearly 40,000 neurons. By aligning the calcium signal of each neuron to three task events-the Go cue triggering the reach, the onset of paw lift, and the contact between the paw and the target-for different target positions, the authors identified different response patterns distributed differently across cortical areas. They defined a set of features that describe the neurons' response pattern, representing the temporal dynamics and tuning properties for the different target positions. These features were used to construct cortical maps, and the authors show that, interestingly, gradient maps obtained from the first derivative of the feature maps reveal sharp discontinuities at the boundaries between anatomically defined cortical areas. Using dimensionality reduction of the neuronal response features, the authors found that, despite clear differences in their average response properties, individual neurons from the same cortical areas do not form distinct clusters in the reduced-dimensional space. In fact, most areas contain heterogeneous neuronal populations, and most neuronal populations are present in multiple areas, albeit in different proportions. Interestingly, the authors identified four neuronal subpopulations based on the distance between the components of the Gaussian mixture model used to model the distribution of neurons within each area. One of these subpopulations is almost exclusively represented in the anterior M2 cortex, while another is broadly distributed across the different areas.

Strengths:

This article is based on an impressive dataset of nearly 40,000 neurons covering a large portion of the sensorimotor cortex and on innovative analytical approaches. This study is likely the first to clearly demonstrate boundaries between cortical areas defined based on the responses of individual neurons. This innovative approach to functional mapping of cortical areas potentially opens up new perspectives for higher-resolution mapping of frontal cortical areas, using a broader repertoire of sensory and motor evoked responses.

Thank you!

Weaknesses:

The second part of the article, which presents multimodal responses in the cortical areas, seems to be a perhaps overly complicated way of showing what has already been demonstrated in numerous recent publications, but these new analyses expand upon these previous observations by revealing an interesting functional organization of the sensorimotor cortex, highlighting interesting similarities and differences between certain areas.

We understand the concern: a number of recent papers have also noted different neuron response characteristics distributed throughout the motor system. We compare and contrast in greater detail following the more specific comments on this below, but we briefly summarize here. The way previous work handled the data – for example, starting with PCA – mixes what neurons are tuned for and when they are tuned for it with what we refer to as the “response format”: properties like tuning sharpness, response duration, etc. We focused primarily on this response format, and designed our features to be mostly independent of tuning preferences or peak response timing. We therefore pick up on different properties of neurons’ responses than those prior works. In addition, no previous work we know of examined these properties across large swathes of cortex at single-cell resolution in the context of forelimb control. Together, these aspects of our work allowed us to produce high-resolution mapping of response properties in a way we have not seen in any prior work.

**Recommendations for the authors:**

**Reviewer #1 (Recommendations for the authors):**

In addition to addressing the weaknesses stated above, I suggest the authors also consider the following.

The one big question left unresolved here is whether we should be thinking about these four subpopulations as distinct types with a biological basis and importance, or just reflections of activity pattern heterogeneity. The authors say that "we did not observe tight clusters in feature space separated by gaps," but their discussion here is light and a bit unclear, and their engagement with the issue of types versus heterogeneity, in my view, could be improved. We do not need "gaps" where the density goes to zero in parameter space, but we do need reproducible troughs between peaks. The authors should clarify if there are substantial and reproducible troughs in the parameter space between their four subpopulations.

This is a great idea, and we have added three analyses and additional text to address it. We break this concern down into two more specific questions, based on the next comment by this reviewer.

(1) Are the clusters well separated / do they have troughs between them? (Note that even with troughs, clustering might not be stable if the clustering algorithm is poorly matched to the shapes of the clusters.)

(2) Is the clustering stable? (It can be stable even without troughs, if, for example, the distribution has a long tail and a GMM needs one Gaussian for the body of the distribution and a second for the tail.)

First, to directly address the presence or absence of troughs between clusters, we have added Figure 9-figure supplement 2A and 2B. For each pair of subpopulations, we trained a logistic regression classifier to separate the 5D feature vectors of the neurons in one subpopulation from the feature vectors of the neurons in the other subpopulation, then projected the feature vectors onto this axis. Note that because the subpopulations are defined by GMMs, which have nonlinear boundaries, the (linear) logistic classifier does not typically produce perfect classification. Nevertheless, this analysis provides a window onto how well separated each cluster is from each other cluster in feature space. In 5 of the 6 pairwise comparisons, it is obvious that the distributions are different and have at least some dip in the distribution density at the boundary. The one pair of clusters without a trough between them were the forelimb somatomotor and hindlimb somatomotor subpopulations. This was surprising to us, given that their likelihood maps are so strongly distinct, but this presumably reflects trying to capture a nonlinear classifier boundary with a linear one (see below). Overall, this analysis argues that the clusters do have fuzzy edges that blend into one another, but reflect concentration of mass near the centers of the clusters we identified.

Second, to address the same question with a different nonlinear method, we have added a version of the t-SNE plot from Figure 7 that is instead colored and contoured by subpopulation identity instead of area (Figure 9-figure supplement 2B). Agreement with the GMMs is not a given here either, because t-SNE is a fundamentally different and independent nonlinear transform from that performed by the GMM classification. Nevertheless, the subpopulations were again nicely separated – though not with troughs, possibly thanks to the inherent difficulty of interpreting point density with t-SNE. Interestingly, here the hindlimb somatomotor subpopulation was the best separated from the other subpopulations, supporting the idea that the lack of separation we observed above with the logistic projections was indeed due to a nonlinear boundary. This analysis again argues that neurons are more likely to have features that lie near the center of a cluster, but that the edges of the clusters run into one another. Additionally, this analysis makes clear that treating the hindlimb somatomotor subpopulation as a second cluster can be supported by other analyses, even if not by the logistic regression projection.

Third, to address the question of cluster stability, we have performed random splits of our data, GMM clustered the two halves independently, applied the GMM from one half to the other, and asked how similar the clusterings are using the Adjusted Rand Index. This produced a value of 0.856, which for this sensitive measure argues that the clustering is rather stable (at least for the three clusters that can be found with all data together, which does not include the smaller-in-size Anterior subpopulation). Note that we did not perform this analysis on the more complicated version where we fit a GMM to each area separately then cluster those; in our main analysis, the hierarchical clustering agreed with what we found by eye, but determining the number of clusters for hierarchical clustering is in general very unstable and so we did not have an objective way to determine the “true” number of clusters.

In addition to these new analyses, we note that three analyses we had already included bore strongly on this issue. Regarding separability of the clusters, the fact that our likelihood maps (Figure 9C-F) were quite distinct for different subpopulations argues that we picked up on ‘real’ differences. Second, Figure 9B found that when clustering non-overlapping data – different cells from different areas – we obtained clusters that were nearly identical in their feature distributions. Third, Figure 10E used the clusterings from different areas’ data to create likelihood maps, and found that they were extremely similar. These analyses together argue strongly that we are finding ‘types’ in a meaningful sense; given that we know the areas do have different distributions of properties, if there weren’t types then clustering would yield different clusters for different areas. Given the importance of the question, however, we are grateful that the reviewer encouraged us to find additional ways to make this point!

The original t-SNE plot is beautiful and quasi-fractalic, but it does not show clear signs of four cell types. The single-neuron activity profiles are clearly heterogeneous in very interesting ways, but heterogeneous does not imply a strong or reproducible multimodality that would indicate meaningful cell types. Clustering algorithms will always spit out an answer. If you just have elements uniformly distributed across a parameter space, plus some noise, when you ask for X clusters, you will get X clusters that have different centroids. When you ask an algorithm to cluster without defining the number of clusters, noise can lead the algorithm to produce a particular number of clusters that again will have distinct centroids. The salient question, though, is whether in the present case there is a parameter space in which the clusters are substantially and or reproducibly distinct. Distinct here would mean that peaks in the density across some parameter space are separated by troughs - again, we don't need true gaps. The more substantial the differences between clusters are (again, not the differences between centroids but the prominence of the density troughs between them), the more biologically meaningful the clustering is likely to be. Reproducibility here could be addressed with resampling methods (e.g., how often do two separate halves of the cells produce the same clusters?).

Please see the reply above, which includes our addressing of this concern.

The Introduction is generally good, but it could further develop existing ideas about how function is distributed across cell types and regions. We would like to be able to imagine different answers to the question of how activity patterns are organized that might have divergent implications for how the circuit works. I understand we have very little to go on in terms of data, but I think it would be helpful for readers to be given more of a sense of what *could* be important.

Good idea. We have added such a paragraph to the Introduction:

“To frame possible outcomes, consider that single neuron responses can vary along many dimensions. Cells could differ according to which movements or time periods they are recruited for (tuning), what movement parameters their activities reflect (encoding), or how their responses are structured across different movements (e.g., nonlinear encoding structure). Further, differences in these response properties across cells could be distributed over the cortical sheet in a variety of ways. Cells could form distinct “categories” or clusters that are spatially well-aligned to the boundaries of anatomically defined regions. Or, categories of neurons might span area boundaries in spatial footprints that do not relate obviously to area boundaries, and that either abut or overlap. At a fine-grained scale, cells with similar responses could be physically located near one another as in primate and feline visual cortex, or similarly-responsive neurons might be salt-and-pepper intermingled as seen in rodent visual cortex or in primate motor cortices during reaching behaviors.”

It should be clarified in the Results how the cue relates to the target location. Most would assume a different cue for each location, but this does not appear to be the case. The authors should clarify whether there was some amount of searching for the precise target location after the reach, or else how the block structure or other sensory information allowed mice to learn where exactly the target would be. In the absence of target-specific cues, some sense of how the mice achieved target-specific reach trajectories should be offered.

Related to this, in Figure 1, it would be good to see some individual trajectories, as they all overlap near the target in the current plot. Clearly, the reaches were targeted, but it is unclear how targeted. Some of the adjustments at the end may reflect searching or palpation to resolve the precise spout location. It is very much ok if the mice were not reaching with micron precision each time to each of 15 different targets, but it would be good to provide the reader a better sense of what the mice were doing.

These are important points. First, to clarify, the Cue is just a Go cue, and was the same for all targets. It is now described in the Results as “non-target-specific”. For additional explanation about supplemental analyses to assess “aiming”, see replies to Reviewer #1 Public Review comments above. Finally, regarding how the mice locate the target: we just don’t know. As discussed above, Galiñanes and Huber found evidence for the mice using stereo sniffing, but whisking, listening to the motors, or some other strategy are also conceivable. We simply don’t have data to weigh in on this. We now make this limitation clear where we describe the task.

In Figure 1A, CFA does not look well aligned with Tennant et al. (2011). CFA should only extend to +1 AP. The overlap of CFA and RFO seems strange. RFO also does not totally align with the injection coordinates used in An et al, biorxiv 2022.

Thank you for your attention to these points. Our designation of the name CFA to the red dashed outline in Figure 1A was consistent with an earlier version of our previous work (Grier et al 2026) wherein we referred to the anatomical outline “MOp-ul” from Munoz-Casteneda et al 2021 as CFA. We have since revised that nomenclature to now refer to the outline as M1-fl, or the forelimb representation of primary motor cortex.

Our placement of RFO was obtained by aligning the Allen CCF from Figure 1K of An et al 2022 to our version of the Allen CCF and outlining the hotspot of RFO with a circle. We have slightly adjusted the location of RFO posterior and medial to more closely align with the injection coordinates reported in the methods of An et al. 2022 of “1.5-1.88 mm anterior from Bregma, 2.25-2.63 mm lateral from the midline.” Because (as far as we understand) the injection coordinates and the map are not perfectly in register, we show a compromise between the two.

We stress that the Figure 1A map is meant to be descriptive in its illustration of the variety of organizational zones that have been identified across mouse sensorimotor cortex.

Discrepancies in the alignment procedure, animal strain, and mapping modality all introduce heterogeneity across mapping attempts that we do not aim to reconcile or resolve here.

Related to this, aspects of the results do seem consistent with the distinction between RFA and CFA, but this is not acknowledged or discussed. For example, the barriers in Figure 6H that lie along the M1/M2 border - these seem consistent with the gap between RFA and CFA. The same could be said for the dim trough along the M1/M2 boundary that appears to separate RFA and CFA in Figure 3B. A slightly more rostral and lateral location of CFA compared to Tennant's definition or the regions backlabeled from cervical spinal injections (see Wang, Maunze et al. J Nsci, 2018) could be expected if flattening the brain under the coverslip for imaging effectively stretches the ML axis, and Bregma (notoriously hard to define reliably at this spatial scale) was defined a bit more caudally here than in other studies. Related to this, it would be better for the field if people described their method for defining Bregma in the Methods. I suggest the authors do this here.

We appreciate the suggestion and have acknowledged the suggested correspondence in the discussion. Given the difference in our approach from those that originally characterized RFA (through ICMS and deep layer projection tracing) we have avoided making overly strong conclusions about this correspondence in our data. See the quoted text below.

“The spatial distribution of modulated cells in Figure 3 suggests a distinction between the caudal forelimb area (CFA, involving M1 and S1-fl) and the rostral forelimb area (RFA) in M2, while the feature gradient boundaries suggest a distinction between M1 and M2 more generally. The absence of a clearly delineated RFA was surprising, given its distinct projection patterns (Carmona et al. 2024; Hira et al. 2013b; Wang et al 2018) and functional differences from CFA (Kristl et al. 2025; Morandell and Huber 2017; Saiki-Ishikawa et al. 2025), but our results might suggest that the activity in layer 2/3 of RFA does not differ markedly from other nearby subregions of M2.”

Regarding bregma, we did not use it for atlas alignment here. Alignment was accomplished through a combination of paw vibration mapping and the location of the central sinus. Bregma’s location was only relevant for our injection of tdTomato labeling, and that labeling was used here only to stabilize the image plane. We include an estimate of it on the map solely in an attempt to be helpful, but we cannot claim we have the most reliable method for defining it.

The authors focus on activity aligned to cue timing. This is sensible, but it could be meaningful to know how this choice affects the definition of organization. If response clustering is largely different across time, it would seem important. I understand that addressing this question may be beyond the scope of this paper. I just wanted to raise the issue with the authors for their future consideration.

We agree that this is important to address directly. There are two aspects to this comment: (1) does it matter if activity from approximately the same time period is aligned to the paw lift or contact instead of the cue? (2) What changes if we use data from a different period of time?

Regarding the first question (alignment), if we switch to aligning our data based on lift or contact, we have more statistically modulated neurons (see Figure 3C), but everything else is qualitatively similar with one exception: the GMM optimization doesn’t separate out the Anterior subpopulation from the Forelimb Motor subpopulation. The Anterior subpopulation only has a relatively small number of members, and they mostly exhibit the strongest peaks in their PETHs when Cue-aligned, so this makes sense. We now show the modulation maps for all of the locking events (Figure 3-figure supplement 1).

The issue of the time window is a little more complicated. There are many choices we made in this work, of course, not least of which are the task we used and the features we chose based on hand-inspection of thousands of PETHs. As we noted in the Discussion, different tasks or different features would likely distinguish more subpopulations from one another. We think of the time window as a feature choice, albeit an implicit one. We chose not to include later time points because this begins to strongly include reward signals, which are known to be large (Levy et al 2020) and can dominate other aspects of the responses. The largest differences we noted when trying time windows that extended later are that mouth-related areas are separated out in the subpopulation analyses, perhaps because of later licking/consummatory responses, but we have not explored fully enough to speak confidently on this point without much more work and another 10 figures. To keep the scope of the paper manageable, we now call out this choice explicitly (see text below). We thank the reviewer for raising these important points.

“Crafting additional PETH features, or using end-to-end neural network approaches to discover other features, might enable the discovery of additional structure (Minderer et al. 2019; Wang et al. 2023b). For example, our PETH features were chosen to be invariant to the onset time of activity, but these onset times were markedly later in lateral M1 than in adjacent M2 or S1-fl. Including onset times, using a wider window of time that includes more of the reward/licking period, aligning data to other behavioral events, or adding other PETH features would presumably result in finer subdivisions of sensorimotor cortex.”

The map in Figure 4 is very cool, and the spatial structure is quite striking. In terms of the actual values of the onset times in each region, I am a little concerned with a dependence on the level of reach-related activity modulation, especially relative to the level of background activity (potentially related to posture). Less reach-related activity and more background activity, which we might expect for trunk and hindlimb regions, could seemingly skew the onset times earlier. We could be getting the right answer, or an answer that makes intuitive sense, for the wrong reason. Can this potential confound be excluded with some sort of control analysis?

The previous text wasn’t clear. We have now clarified what we meant, very much in line with the reviewer’s thoughts. In addition, note that our change to what is displayed in the histogram (now neurons, previously pixel values) makes clearer that there is a multi-peaked distribution of onset times and it is mostly the prevalence of each peak in each area that varies. The text now reads:

“These distributions over neurons revealed clear differences in the overall profile of activation: early onsets were more prevalent in S1 trunk and hindlimb regions, perhaps due to activity related to the animal stabilizing itself even if the neurons became more active later; then M2, and finally S1-fl and M1. Nevertheless, each area contained neurons activated at any given time in the trial.”

The "Peak time variation" metric could potentially vary with activity level, with lower, noisier activity levels making cells appear less persistent. Perhaps a control analysis, based on SNR or some reasonable assumptions of the linkage between calcium signals and spiking, could be performed to measure the extent to which this could be creating differences between regions.

Good idea. We have now performed this analysis, and the reviewer was correct: the correlation between peak time variation and a simple metric of SNR (assessed as range of PETH / max s.e.m.) was substantial: ⍴=-0.53. We now report this correlation and describe in the Results that this metric is driven by both true peak time variation and trial-by-trial variation. Thank you for this!

“Peak time variation. To quantify whether a neuron’s firing peaked at the same time for every target or varied by target, we found the peak firing rate of the response to each target, then computed the standard deviation of these peak times across targets. This value is therefore higher if the peak time varied and nearly zero if the timing was consistent. Notably, this measure correlated substantially with overall signal-to-noise ratio of a neuron’s PETH (Spearman’s ⍴=-0.53; Methods), and thus partly measures trial-to-trial variability, not just true peak timing variability. This metric was quite low in M1, indicating highly consistent timing of the activity peak (and reliable responses), and was highest in the posteromedial part of M2 (presumably corresponding to the hindlimb representation) and the posterior tip of S1-hl (Figure 5B).”

One could argue that the likelihood calculations illustrated in Figure 8 are biased higher for neurons within each region since they were used for defining the likelihood for that region. I think these likelihood calculations should be done for separate neurons other than the ones used to compute the mixture model for each region.

We agree with the point about bias: the by-area GMM in Figure 8 is biased toward cells within the area, though the effect is probably quite mild given the large numbers of neurons and modest number of parameters. However, this model was intended to make the point that even if you give an area an unfair advantage, you still can’t cleanly isolate it. This was intended to help motivate the following analysis of subpopulations, and we have now made this logic clearer. Doing it this way has the advantage that the GMM components are identical between Figures 8 and 9, while if we held out the test neurons it would not be possible to make them the same without some complicated version of bagging on the GMM components. The reviewer is right that we should make this bias explicit, though, and we have now done so:

“This mapping approach is explicitly biased toward finding feature differences between areas, allowing for a direct test of the hypothesis that response profile distributions are area-specific.”

To me, the last Results section (Spatial overlaps between subpopulations indicate intermingled members) does two things: it shows you get the same results when you map each cell to a subpopulation independently of its area, and it shows that defining the subpopulations with cells from each area gives you essentially the same results, arguing against spatial variation of properties within subpopulations. I worry that these two points are getting merged together or not made clearly enough here, especially the first one. In general, the logic of this section does not seem well conveyed.

Thank you for the feedback. In particular, your first point is made by Figure 9-figure supplement 4 when we fit an area-agnostic GMM to all modulated cells in the five main areas. However, your second point is one of the two main goals of the last Results section, along with the demonstration of the spatial distributions of cells after hard-clustering them by subpopulations. We have tried to clarify these main points further through substantial edits of the results section for Figure 10.

One set of ideas that is highly relevant and should be raised concerns an ethological organization of the motor cortex. Since the observations of Graziano, there has been a steady stream of results describing ethological organization in rodents as well. This literature is briefly reviewed in Kristl et al., Nature Communications, 2025. For example, because of the potential for a differential involvement of grasping movements across different target locations, some of the variation in neuronal tuning described in the present manuscript may stem from a region preferentially involved in grasping.

We agree that the Graziano literature, and the substantial literature in rodent that was inspired by Graziano’s work, is highly relevant to understanding the organization of motor areas. Kristl 2025 handles these issues very thoughtfully. The challenge here is that there are many possible different reconciliations of the stimulation results with ours, and some seriously unresolved challenges in doing so. To name a few:

Our subpopulations and high-gradient boundaries both give quite different pictures than microstimulation does in rodent motor and sensory cortices. In particular, microstim produces more subregions that evoke different movements than we identify, and the borders don’t generally line up. This implies that the mapping between the two approaches is probably complicated.

There is a completely alternative possibility to explaining the Graziano-like results: microstimulation is thought to preferentially hit axons, and some of these projections reach the medullary motor regions. Given that the medullary motor regions have known topography in the movements they evoke (Yang et al 2023) – but may or may not be driving the movements during flexible behavior – the two approaches may not be reconcilable. Or, it may require a much deeper understanding of medulla as driving the primary movement and cortex acting as a residual controller. This is an exciting set of ideas, but as yet very underdeveloped in our understanding.

We don’t know if the subpopulation structure exists at all in L5, or in the PT cells, and if it does whether it differs. This is crucial given the frequent targeting of deep layers by ICMS stimulation protocols.

As we caution in the Discussion, it is possible that our subpopulation findings are at least partly specific to the task we used.

Although it is beyond the scope of this paper and will be addressed thoroughly in separate work, we have spent significant time with encoding models for joint angles and high-level target encoding in these same data. Given those results, we are fairly confident that the reviewer’s reasonable guess, of tuning variation due to intersections between body parts, does not seem to be the main driver of the subpopulation structure we find.

After careful thought and discussion amongst the authors, we did not think that including this discussion in the paper was likely to improve interpretability of the present results for most readers. We very much agree with the point, though, and when we can narrow down the possible explanations in the future (likely in our next paper on this topic, which will address encoding) we plan to address it. We thank the reviewer for encouraging us to think through this.

Minor:

(1) Page 3: "densely shared" - perhaps "broadly shared"? Dense implies most/all the neurons get the same signals, which may not be true.

Changed to “widely”.

(2) Page 4: "data-driven approaches" - could be more specific - isn't everything we do data-driven?

Changed to “bottom-up”.

(3) Page 4: "spanned areas" - perhaps "spanned multiple cortical areas", since everything spans an area.

Changed to “spanned multiple areas” (we mention cortex just a few words earlier).

(4) Page 5: "intervals were generally fast" - awkward, "short" perhaps.

Agreed, changed.

(5) Page 5: "which asks whether the activity for a neuron changes over time consistently in relation to any target" - Rephrase to disambiguate between consistent temporal variation in firing for all targets and variation across targets in the firing patterns. In other words, are we talking about cells that are just modulated during reaching, or cells whose firing patterns differ across targets?

Changed ending to “to any given target”. The ZETA measure really does simply ask whether there is a change in firing rate over time that is consistent across trials, for each target independently. A neuron that exhibits an identical bump for all targets would register as modulated. We chose this measure in part because of the number of temporally-modulated but untuned cells. This wasn’t very clear as we had written the text, so we now note this explicitly in the Methods. Thank you for pointing out that this wasn’t clear.

“For all analyses, only neurons modulated by the relevant locking event were included. Note that this measure looks for modulation over time to any target; it is indifferent to whether the neuron exhibits tuning across targets.”

(6) Figure 1: It seems like some of the abbreviations used in 1A have not been defined yet in the paper.

Yes. It’s a long list, and we wanted to put the citations for the description of each area together with the definition of the acronym. Moreover, we wanted all this info together with the description of how we aligned these area descriptions from others’ work with one another on the Allen atlas. This was impractical in the caption, and would be a long digression for what is intended as a simple point in the Results, which is why we refer to the Methods here.

(7) Page 8: "Given that these areas have known spatial organization within them and structure was apparent by eye in the spatial scatterplot of modulated neurons (Fig. 3A)," - it is not clear what spatial structure we are supposed to see in 3A.

Good point. We have changed the parenthetical to: “(for example, the less modulated band along the M1/M2 border in Fig. 3A)”

(8) Page 8-10: The region-wide onset analysis breaks up the flow from PETHs to the metrics used to quantify them. I suggest moving this section (Onset of neural activity varied with somatotopy and subregion) to later in the manuscript.

We appreciate the reviewer’s input on organization. We went back and forth many times in how to organize the many results in this paper. The reviewer is right that this analysis breaks the flow, but the reason we included it where we did was threefold. First, it uses an easily-understood metric to introduce the reader to how we made maps from single-neuron features. Second, it easily introduces the power of making such maps. Finally, it makes clear that if we are not careful with how we handle time in the feature design, timing will dominate.

All these things said, this has helped inspire us to add a result in which we re-examine timing broken down by subpopulation (Figure 9-figure supplement 2C). It shows that subpopulations timing distributions appear more distinct than distributions for areas, but there is still substantial heterogeneity in timing that is explained by location in cortex and not subpopulation membership alone.

(9) Page 12, Target tuning linearity: This metric should be clarified in the Results. It is not clear how the 2D of targets is turned into 1D. Also, the plot in the figure has correlation on the y-axis, and it is not clear how each target location gets its own correlation value. The phrase "optimized anchor target" is unclear.

Agreed this needed to be clearer. The text in the Results now reads: “To quantify how linearly a neuron’s activity related to target location in physical space, we correlated the 15D vector of mean activity of the neuron for each target with the 15D vector of the targets’ ordinal distances from the neuron’s preferred target (Methods).” In agreement with your suggestion, we have dropped use of the phrase “anchor target” in favor of “preferred target”, which should be clearer. We have also revised the Methods text accordingly to clarify.

To directly answer your question, we turn the targets from 3D positions into 1D by computing the ordinal distance of each target from a preferred target. (Note that the preferred target is actually the one that maximizes the resulting correlation; this is detailed in the Methods). There therefore aren’t 15 correlations; we’re correlating two 15D vectors, where each has one element per target and the “ordinal distance” vector has a zero for the preferred target. Hopefully the new description makes this clearer.

The figure schematic was unclear, thank you for catching that. We have updated the Y axis to read “mean activity” and the X axis now reads “dist. to pref. target.”

(10) Page 12, paragraph beginning "We also compared our metric maps simply using the top 20 PCs." - This paragraph is unclear, since both sentences refer to using the metrics. I would guess the authors mean that the metric maps were compared with and without PCA and basis rotation, but this is not clearly stated.

Thank you, this was unclear as written. We have changed it to:

“We also compared our metric maps with maps generated from the top 20 PCs of the PETHs (Methods), rotated using VARIMAX to identify a sparser basis (Musall et al. 2019).”

(11) Page 18: "These results make clear that the working hypothesis - of areas with well-separated feature distributions - is incorrect." This is the clearest statement of the impact of the results. The authors could consider including this in the Abstract or Introduction.

Thank you for pointing this out. We agree, and have added a similar phrase to the Abstract.

(12) Figure 9: It would be great to also just see the average PETHs for each of the four clusters to get a better sense of how their time series differ.

Good idea. The feature computations are a many-to-one mapping, so it’s not possible to literally generate a PETH from the mean of the cluster, but we have added PETHs from well-modulated neurons that are near the means of their subpopulations (Figure 9-figure supplement 1).

(13) Figure 9B: Colorbar has no label.

Fixed, thanks.

(14) Figure 9C: Need a colorbar - need to see the difference in density for locations.

The color map is the same Figure 8B, which is now noted in the caption for Figure 9C. The scaling of likelihoods is almost totally uninformative; they’re not well-behaved like probability distributions, so you’ll note that even on Figure 8B the labels are simply “max likelihood” and “min likelihood”. The important pieces of information here are that these are log likelihoods (noted in the Figure 8 caption), and the visualization of the color map itself (from the color bar). Given these considerations, we have elected to keep the maps themselves a little larger by not trying to squeeze in a minimally-informative colorbar to all of the plots, but thank you for noting that the reference to 8B was needed.

(15) Page 22: "additional spatial structure could be present" - The nature of the additional spatial structure here is a bit opaque. The authors could clarify what additional structure may be present.

Good idea. This paragraph now reads:

“The overlaps in the subpopulation likelihood maps above imply that members of different subpopulations are spatially intermingled, but it is less clear whether each subpopulation has homogeneous response profiles across space. In particular, the use of likelihoods mixes two properties: the fraction of neurons in a given neighborhood that are members of each subpopulation, and the heterogeneity of response profiles amongst members of that subpopulation. These properties could vary systematically with respect to one another, and the spatial structure shown by the likelihood map does not disentangle them.”

(16) Figure 10E, legend: "GMM component" - I think this should be "GMM subpopulation" to avoid confusion with the previous use of "component" above, referring to the components of the GMM models for each region.

Thank you – good catch. Changed to “Likelihood map”.

(17) Page 24: "Note that this consistency also validates the use of clustering to combine components and identify the subpopulations in the first place." - I don't totally get this, and how this result validates the method of combining components, as opposed to just clustering all the cells from all regions at once. Perhaps the implied opposing strategy is not clear here.

We have changed this sentence to:

“Note that this consistency mirrors the low Bhattcharyya distances between corresponding GMM components in Figure 9B, and further validates the use of clustering to combine components from different areas.”

Regarding the reviewer’s larger point, we have three thoughts. First, we do also show the result of fitting the GMM to all cells together (Figure 9-figure supplement 4).The result is similar, but the Anterior subpopulation is lost because its membership is low and so the ICL criterion can’t justify a fourth cluster. Second, because we imaged more neurons in some areas than others, fitting the GMMs to each area separately put their representations on a more equal footing. Finally, doing the analysis this way allowed us to most directly compare our two hypotheses, as illustrated in Figures 8A and 9A.

(18) Page 25: "in the zones where different subpopulations overlapped" - I would omit this, since "intermingled" seems to mean exactly this.

We included this phrase to prevent quickly-skimming readers from incorrectly concluding that the subpopulations overlapped entirely and were therefore intermingled everywhere. The reviewer is right that it’s unnecessary for a careful reader, but we aimed to prevent misinterpretation by readers that might skip to the Discussion for a results summary.

(19) Page 25: "content of the activity, but also its format" - the difference between content and format is not entirely clear. Metaphor not quite metaphoring here. Agreed. We have added examples to clarify.

“This makes clear that there are potentially important differences not just in the content of the activity (e.g., encoding target vs. movement commands (Grier et al. 2026)), but also its format (e.g., linear encoding vs. nonlinear, persistent vs. brief responses).”

(20) Page 30, bottom: In the description of the behavior, more details should be provided, especially since the paradigm is new. For example, it says the block size was reduced - what was the ultimate block size?

Targets were cued randomly in the behavior performed during neural recordings. Blocked trials were used during training and were phased out incrementally as performance improved. This and various other details have been added. Please let us know if there are other specific details you would like to see in the final version.

(21) Page 39, citation of An, Mulcahey et al.: There is a biorxiv version with a different author list that could be cited.

This was an error with our citation manager, and has been corrected. Thanks for catching it.

**Reviewer #2 (Recommendations for the authors):**

Overall, this is a remarkable study with well-designed in-depth analyses, and I only have some minor suggestions that could help improve the clarity of the paper.

Thank you!

General:

It is not immediately clear to me why the GMM approach used in this study is more interesting than a clustering approach based on single-neuron response patterns (See Esmaeli et al., Neuron 2021 or Oryshchuk et al., Cell Report 2024). But my impression is that it led to the same observation that most clusters are widely distributed across cortical areas, with different proportions, but a few clusters are quite specific to a few areas. A noticeable difference perhaps is the number of clusters - or response profile - that seems particularly low (only 4) in the current study. Could the authors clarify and comment on that, maybe?

The reviewer brings up an interesting point: at heart, these works ask related questions, albeit about different effectors, tasks, recording modalities, and types of information encoded. Those differences probably mean that results cannot be directly compared, but we can certainly discuss the methodological tradeoffs. The two papers mentioned take a more traditional first step, using PCA on the vectorized PETHs to reduce dimensionality, then layer on a spectral approach to improve clusterability. These are good methods; we use something similar as our alternate method, applying VARIMAX to the PCs instead of spectral methods to preserve linearity of transforms. For the kinds of responses both they and we have, PCA will tend to most strongly pick up two aspects of the responses: tuning and timing. This is because vectorized PETHs will have large values in the rows corresponding to the target/condition and time points where the high activity is, and the alignment of these profiles with those of the other neurons will capture a large fraction of the variance. For data like either theirs or ours, this would tend to cluster apart left-tuned cells from right-tuned, and (more importantly here for revealing spatial structure) early-response cells from later response cells. That intuition is consistent with what those papers report, and examining our VARIMAX’ed PC plots closely (which have sharpened in the latest version thanks to improved normalization), we can see that they break apart sub-regions largely based on timing. In our feature approach, we intentionally chose our features to be largely invariant to both tuning preferences and timing. Instead, we chose our features to pick up on what we call the single cell “response format”: response duration; peak time variation (but not absolute timing); and tuning sharpness, persistence, and linearity. These different methods pick up on different aspects of responses.

To double-check that the PCA-then-spectral approach reveals similar structure to our use of VARIMAX on the PCs, we tried applying the suggested method to our data. We applied spectral clustering to the N x 20 PETH PC feature matrix, then fit an area-agnostic GMM to the spectral features. We plot the likelihood map for the components of a GMM with 10 modes. The GMM components did not display clear spatial structure beyond that observed in the VARIMAX’ed PCs (Figure 5-figure supplement 1) and were less interpretable than those identified by area-agnostic clustering of our response features (Author response image 1). As noted, the number of subpopulations identified by the clustering of our hand-engineered features is lower than what would be obtained from clustering the PCs of the PETHs. This is likely the result of the substantial heterogeneity in activity onset and preferred target that is preserved by PCA. Because our central approach is largely agnostic to these two sources of variation, the number of identified clusters reflects the dominant patterns of variation beyond these two sources.

**Author response image 1.** GMM fit to spectrally transformed PETH PCs, agnostic to anatomical areas. One GMM was fit to the spectrally-embedded PC feature vectors of cells from all 5 main areas. Each component of a 10 component model is shown.

Also, I think it would greatly help the reader to return to PETHs at some point, if possible, to show the response profiles of each identified neuronal subgroup (page 20). To what extent are they similar or different across the cortical areas (for the same neuronal subgroup)?

This is a good idea. We have added a figure to address this question and the related question by R1 (Figure 9-figure supplement 1). In short, given the wide variety of PETHs we observed, there is of course still substantial variation within subpopulation, and some mild but systematic differences in the distribution of what we observe across areas. We now discuss the conclusions from this plot in the Results:

“As a qualitative depiction of the response profiles identified with each subpopulation, we plotted the two highest-likelihood cells for each area/subpopulation combination (Figure 9-figure supplement 1). These examples reveal stereotypy in the subpopulation responses across areas, but also show variation across areas, especially for the two somatomotor subpopulations.”

Specific:

(1) Figure 2B and M&M: the 3D spatial organization of the target locations is not immediately clear. What is the spacing between target locations? What is the 'final azimuthal spacing'?

Added, thanks. The pairwise horizontal distances between targets were between 1.72 and 6 mm apart and the vertical spacing within a column was 1 mm. “Final azimuthal spacing” just referred to the targets being closer together during training and our gradually spacing them apart to their final locations. We have also added some relevant details about the training.

(2) Figure 2C: It would help to have a scale bar (mm).

Added, thanks.

(3) Figure 2C: It would be easier to appreciate the variability of the trajectories across trials to plot an overlay of trajectories to one target only (could be a Supplementary Figure).

The reviewer has a good point: the variability and accuracy of aiming was hard to ascertain from the plot. We experimented with a few options for making this clearer most effectively. We have now added Figure 2-figure supplement 1 that shows in the third subpanel of panel A the finger centroid trajectories for one of the 15 targets highlighted for the mouse shown in Figure 2C, mouse 3. The centroid trajectories for all other mice are shown as well to illustrate similarities and differences across animals as well as the overall variability. As noted elsewhere we have also included an analysis of the variability of the centroid trajectories, showing that reaches to a given target were more similar than reaches to different targets. We think this provides a fuller picture of the behavior and intend to provide still more detail in future work. Thank you for suggesting additional detail here!

(4) Figure 4: It would be nice to also show the amplitude-normalized grand-average PETHs for the different areas.

This is an interesting suggestion. After careful consideration, we think that this analysis is not as effective for depicting overall timing and modulation profiles as the current ones, given the strong amount of target selectivity and response time heterogeneity (now better visible in the revised Figure 4A). When computing the grand mean of all cells within each area, the dominant features distinguishing areas are onset time and response duration. The differences across areas in these two features are better supported by the analyses of Figures 4 and 5 due to the large amount of heterogeneity in responses within each area. We thank the reviewer for encouraging this exploration; more complicated spin-offs will likely inform additional timing analysis in the next paper on these data.

(5) Figure 7C: figure legend - although it is quite self-explanatory, please explicitly indicate which pattern corresponds to the 'Three contour levels (98%, 95%, 90%)'.

We have now added this as a legend on the figure panel itself (here and on similar plots). Thanks for pointing this out.

(6) Figure 8: Is there also an interesting asymmetry between sensory are motor areas, with neurons in sensory areas being more likely associated with motor areas (B and C), whereas neurons in motor regions are less likely to arise from the distribution of sensory areas (dark blue color in frontal regions in D, E, and F)?

This is an interesting observation, but we understand it to be an artifact of colormap scaling. As mentioned above, likelihoods are not well-behaved like probability distributions are: for example, they are not bounded at 1, and their sums over a dataset can have any positive value. The only things that can be interpreted are their relative values. This makes their scaling functionally arbitrary – you’ll notice we used “min likelihood” and “max likelihood” instead of numbers, which would be nearly meaningless – and therefore presents a problem for scaling the colormaps. We don’t know of a principled way around this problem. To deal with it, we simply put the ends of our colormap at the extreme pixel values. It so happens that both the M1 and M2 maps had a handful of neurons in a less-sampled spot at the bottom of M2 that were very low-likelihood, which results in what you noticed. We debated removing those neurons for this purpose, but we had no basis on which to do that kind of manipulation, so we left it as the most honest representation of the data we could produce.

To clarify this, we now mention in the caption “The ends of the colormap were set to the maximum and minimum likelihood values for each map.”

(7) Figure 9B: there are two-time 'S1-hl: 1' indicated at the two bottom rows of the distance matrix. I suppose one of them should be 'S1-tr: 1' instead?

Fixed, thanks for catching it.

(8) Page 20: 'This hinted at a second hypothesis: that some of the 'modes' (groups of neurons) discovered separately in each area might correspond.' ???

We had meant “mode” as in “multimodal”, but it was very unclear. We have rewritten the sentence:

“This hinted at a second hypothesis: that a peak in the multimodal distribution from one area might correspond to a peak in the multimodal distribution of a different area.”

(9) Figure 9S2: Please indicate for which area each map is computed.

The caption was not clear enough about what we were doing here: we fit the GMM on all neurons together, ignoring which area they came from. We have now clarified it in the caption:

“One GMM was fit to the feature vectors of cells from all 5 main areas. Each map plots the likelihood for all cells to each of the three components of this area-agnostic GMM.”

(10) M&M, Subjects and surgical procedures: 'ambient temperature of 71.5 {degree sign}F', please use international units.

Done.
